# Defective interfering influenza virus confers only short-lived protection against influenza virus disease: Evidence for a role for adaptive immunity in DI virus-mediated protection in vivo

**DOI:** 10.1016/j.vaccine.2011.06.114

**Published:** 2011-09-02

**Authors:** Paul D. Scott, Bo Meng, Anthony C. Marriott, Andrew J. Easton, Nigel J. Dimmock

**Affiliations:** School of Life Sciences, University of Warwick, Coventry CV4 7AL, UK

**Keywords:** Influenza, Defective interfering, Immunity, Protection, SCID

## Abstract

We have shown earlier that a single dose of cloned defective interfering (DI) influenza A virus strongly protects mice from disease following a lethal challenge with different subtypes of influenza A virus. These animals suffered no clinical disease but experienced a subclinical infection which rendered them immune to reinfection with the same challenge virus. However, little is known about how DI virus achieves such protection. Here we investigated the role of adaptive immunity in DI virus-mediated protection using severe-combined immunodeficient (SCID) mice, which lack competence in both B- and T-cell compartments but retain NK cell activity. SCID mice which were treated with DI virus and infected with influenza virus initially remained completely well, while infected litter mates that received UV-inactivated DI virus became seriously ill and died. However, after 10 days of good health, the DI virus-protected SCID mice developed a clinical disease that was similar, but not completely identical, to the acute influenza disease. Disease was delayed longer by a higher dose of DI virus. We excluded the possibilities that the DI virus load in the lungs had declined, that the DI RNA sequence had changed so that it no longer interfered with the infectious genome, or that infectious virus had become resistant to the DI virus. These data show that while DI virus provides full protection from the acute disease in the absence of adaptive immunity, that same immunity is essential for clearing the infection. This indicates that the conventional view that DI virus-induced protection is mediated solely by competition for replication with the challenge virus is incorrect for influenza virus.

## 1 Introduction

In the course of replication most viruses make defective-interfering (DI) viruses, which are virus particles composed of a normal set of viral proteins encapsidating a deleted version of the viral genome. Because they lack essential genetic information, DI viruses are replication deficient. Replication of the defective genome is achieved by the presence in the same cell of a genetically compatible infectious genome, usually from the virus that generated the DI genome, and which provides the missing function(s) *in trans*. DI virus is thus totally dependent on infectious virus for replication. Interference occurs when the ratio of defective: infectious genomes increases to a level which results in a reduction of the amount of infectious virus produced [1–5]. Most of our knowledge comes from studies in cultured cells, but there is also limited evidence that DI virus can protect against virus diseases in vivo [6–10]. The conventional view, developed by extrapolation from in vitro studies, is that the protection afforded in vivo is also due to competition between the DI and infectious viruses at the level of genome replication. However, in those cases where in vivo protection has been seen there is little direct evidence for this or any other mechanism.

Influenza A viruses have a genome that comprises 8 separate segments of single stranded, negative sense RNA. DI influenza viruses arise readily and their study has a long history extending back over 60 years [5,11–13]. DI RNAs can potentially arise from all viral segments, but are most commonly derived from segments 1–3. All influenza DI RNAs formed from their cognate RNA and contain a large central deletion of approximately 80%, but retain the terminal sequences which control replication and packaging. It is hypothesized that an infectious particle packages one of each of full-length segments 1–8, while the DI virus particle packages a DI RNA in place of its cognate full-length RNA, plus the other 7 full length RNAs. Most DI influenza virus preparations contain many different DI RNA sequences, but it is not known if a single DI particle can contain more than one DI RNA, or if there are other DI particles in the preparation that contain a DI RNA derived from a different segment. The position and extent of the central deletion in the DI RNA is highly variable so that DI RNAs originating from one genomic segment can have many different sequences. For all these reasons it has been difficult to determine the relationship between a DI RNA sequence and the biological properties of the DI virus [14]. We recently solved this problem by using molecularly or biologically cloned viruses that contain one major species of DI RNA [14–18], and subsequently characterized one DI virus, containing RNA 244, that strongly protects mice from clinical disease caused by various influenza A virus subtypes [18]. However, it is not understood how influenza DI virus mediates such protection in vivo. In principle, DI viruses could act in vivo by interfering with the production of homologous virus (as described above), by stimulating adaptive immune responses, by stimulating innate immune responses, or by means as yet unknown. More than one of these mechanisms may operate at any one time. We have shown previously that various aspects of the humoral and T cell-mediated arms of murine adaptive immunity interact with infectious virus in the presence of non-cloned DI influenza A virus. The data showed that the responses to infection were modified in several unusual ways by the presence of active DI virus (see Section 4) [19–25]. Here we investigate how severe combined immunodeficient (SCID) mice that completely lack adaptive immunity but retain NK cell activity respond to a mixture of infectious virus and in conjunction with treatment with cloned DI virus that confers protection from disease in immune-competent animals. SCID mice have been used extensively for investigating the role of the immune system in recovery from influenza virus infections [26–31].

Analysis of the mechanism(s) by which DI viruses prevent disease in treated animals is not fully understood. The DI influenza virus particle and its component proteins are indistinguishable from those of infectious virus and, in principle, they may be capable of stimulating a similar adaptive immunity as to that resulting from exposure seen with to a conventional inactivated influenza virus vaccines. The data show that adaptive immunity is not required for DI virus to protect SCID mice from acute influenza. However, in contrast to immune-competent animals, a delayed onset disease occurred about 1 week later, indicating that adaptive immunity is required to act in concert with DI virus to clear the infection.

## 2 Materials and methods

### 2.1 Production of 244 DI virus by reverse genetics

The 244 DI RNA used here to protect mice was originally generated spontaneously during transfection of 293T cells with plasmids [32] to make infectious influenza A/PR/8/34 [18]. After 24 h, the 293T cells were trypsinized, mixed with MDCK cells and re-plated, and culture supernatants harvested 7 days later. Resulting virus was passaged twice in embryonated chicken's eggs. The resulting mixture of 244 DI virus, packaged in a A/PR8 particle, and infectious helper A/PR8 virus was purified by differential centrifugation through sucrose. Stocks were resuspended in PBS containing 0.1% (w/v) bovine serum albumin, standardized by haemagglutination titration, and stored in liquid nitrogen. Before inoculation into mice, helper virus infectivity was eliminated with a short burst (40 s) of UV irradiation at 253.7 nm (0.64 mW/cm^2^). This is referred to as ‘active DI virus’. The UV inactivation target is viral RNA, and UV has little effect on the DI RNA because of its small target size, 395 nt compared with 13,600 nt for infectious virus. Longer UV irradiation (8 min) inactivated mouse-protecting activity and provided a preparation that controlled for any immune system-stimulating or receptor-blocking effects (‘inactivated DI virus’). However, UV treatment did not completely destroy all DI RNA. UV did not affect haemagglutinin or neuraminidase activities.

### 2.2 Mice

We used wild type C3H/He-mg (H-2^k^) mice (bred in-house), wild type Balb/c (H-2^d^) mice (Harlan UK Ltd.), and mutant Balb/cJHan™Hsd-Prkdc^scid^ mice (Harlan) with a defect in the Prkdc gene which encodes DNA-PK. This leads to aberrant VDJ recombination and hence deficient B and T cells. SCID mice have a normal complement of NK cells. Wild-type Balb/c mice required 2 × 10^3^ ffu of WSN challenge virus to cause consistent but non-lethal clinical disease; this was twice the dose needed for C3H/He-mg mice [18]. Balb/c^scid^ mice were also infected with 2 × 10^3^ ffu of WSN. Adult mice (4–6 weeks old) were inoculated intranasally under light ether anaesthesia as previously described [33,34], with a 40-μl inoculum divided between the two nares. Mice were given various combinations of active DI virus, UV-inactivated DI virus, infectious challenge virus (A/WSN), or diluent. Infectious challenge viruses were titrated in mice to determine a dose for each that caused comparable respiratory disease. The health of mice was assessed clinically and by change in group weight [33]. Clinical criteria were scored as follows: 1 point for each healthy mouse; 2 points for a mouse showing signs of malaise, including some piloerection, slightly changed gait, and increased ambulation; 3 points for a mouse showing signs of strong piloerection, constricted abdomen, changed gait, periods of inactivity, increased breathing rate, and sometime râles; 4 points for a mouse with enhanced characteristics of the previous group, but showing little activity, and becoming moribund; such mice were culled when it was clear that they would not survive; and 5 points for a dead mouse. To allow comparison, the total clinical score was divided by the number of mice in the experimental group. Lungs were scored for consolidation by estimating the percentage of the lung surface that had developed a plum-coloured discoloration. They were stored post-mortem at −70 °C, and later examined for virus infectivity, virion RNA, and 244 DI RNA. Animal experiments were approved by the University of Warwick's Ethical Review Committee and the UK Home Office, and followed the guidelines of the UK Coordinating Committee for Cancer Research.

### 2.3 Quantitative PCR

RNA was extracted from the left lungs of mice by grinding with sterile sand and Trizol (Invitrogen). Quantitative real time PCR was performed on an ABI prism 7000 to quantitate virion-sense (RNA^−^) in infected mouse lung. We used the following primers and probes: segment 1 F (5′ TGCAATGGGACTGAGAATTAGCT 3′), segment 1R (5′ TCCGCTTGTTCTCTTAAATGTGAAT 3′) and probe (5′ VIC-CACCAAAACTGAAGGAT 3′); 244 1F (5′ CATAATCAAGAAGTACACATCAGGAAGAC 3′), 244 1R (5′ CTCTTTGCCCAGAATGAGGAAT 3′) and probe (5′ FAM-CCCTCAGTCTTCTCC 3′); segment 7 1F (5′ CTTCTAACCGAGGTCGAAACGTA 3′), segment 7 1R (5′ GGATTGGTCTTGTCTTTAGCCA 3′) and probe (5′ FAM-CTCGGCTTTGAGGGGGCCTGA 3′) [35]. Primers were synthesized by Invitrogen, and the probes by ABI. To distinguish the 244 segment 1 DI RNA from full-length segment 1, a probe was designed to cover the DI RNA junction region formed when the terminal segment 1 fragments were ligated, and which is absent from full-length RNA. A unique segment 1 probe was designed from the region which has been deleted from 244 DI RNA. A standard for each virion-sense RNA stock was made by subcloning PCR products of either full length RNA or the region flanking the amplicon in pGEMT-easy vector (Promega). RNA was transcribed using the T7 or SP6 RNA polymerase (MEGAscript, Ambion), the mix was digested with DNase I, and RNA purified by electro-elution. After ethanol precipitation, RNA was resuspended into RNase-free water and quantitated on a Nanodrop 1000 (Thermoscientific, Wilmington, DE). Standard curves were generated by performing 10-fold serial dilutions of known RNA copy numbers with each dilution assayed in triplicate. The reaction was conducted at 50 °C for 2 min, 95 °C for 10 min, then 40 cycles of 94 °C for 15 sec followed by 60 °C for 1 min.

### 2.4 Infectivity assay

The right-hand lung from each infected mouse was homogenised with sand in PBS containing 0.1% (w/v) BSA, and centrifuged to remove debris. A/WSN infectivity was titrated in a focus-forming assay using MDCK cells in 96-well plates in triplicate. Cells were incubated at 33 °C for 18 h, fixed in 4% (v/v) formaldehyde, and blocked with 5% (w/v) milk powder in PBS. Virus-positive cells were detected using a mouse monoclonal antibody specific for the A/WSN haemagglutinin, and a goat anti-mouse IgG–alkaline phosphatase conjugate (Sigma), both in buffered saline containing 0.1% (v/v) Tween, and finally incubated with an alkaline phosphatase substrate (NBT/BCIP in TMN buffer; Sigma). At least 50 stained cells (foci) at an appropriate dilution were counted in each of three wells and averaged to give a titre in focus-forming units (FFU)/lung.

## 3 Results

### 3.1 SCID mice are protected from acute influenza by DI virus, but eventually develop a delayed onset disease

**Fig. 1:**
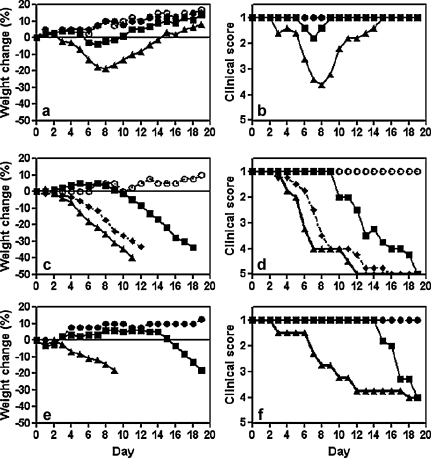
Protection of SCID Balb/c mice by different doses of DI virus. Wild type (*wt*) Balb/c and Balb/cJHan™Hsd-Prkdc^scid^ (SCID) mice were inoculated with inactivated DI virus + A/WSN, active DI virus + A/WSN, A/WSN, DI virus, or diluent. Mice were monitored daily for weight change (left-hand panels) and clinical assessment (right-hand panels). Panels (a) and (b), *wt* mice, and panels (c)–(f), SCID mice. Mice in panels a to d were treated with 1.2 μg of DI virus or inactivated DI virus, and mice in panels e to f with 12 μg of DI virus or inactivated DI virus: (■) active DI virus + A/WSN; (▴) inactivated DI virus + A/WSN; (♦) A/WSN alone; (●) active DI virus alone; (○) diluent. Data are representative of two separate experiments.

Before examining SCID mice we tested the infection parameters of A/WSN in the immune competent Balb/c strain from which they had been derived. Mice inoculated simultaneously with 1.2 μg of active DI virus and infected with A/WSN were either completely protected or suffered only a mild clinical disease of short duration with slight weight loss (Fig. 1a and b). In contrast mice inoculated simultaneously with the same amount of inactivated DI virus and A/WSN lost 19% of body weight at the peak of infection (Fig. 1a); all became seriously ill but then recovered (Fig. 1b). After recovery mice in all groups remained healthy and continued to gain weight with no untoward signs for the duration of the experiment (19 days). Such mice were immune to rechallenge with high dose A/WSN [18] (data not shown). There was essentially no difference in disease progression between mice inoculated intranasally with A/WSN and mice inoculated with inactivated DI virus + A/WSN (data not shown).

SCID mice infected with A/WSN succumbed to a disease similar to that seen in immune-competent Balb/c mice as judged by clinical signs and weight loss from day 3 after infection, progressing to death or to the point at which they had to be euthanized (Fig. 1c and d). The dynamics of disease were very similar in SCID mice inoculated intranasally with 1.2 μg (Fig. 1c and d) or 12 μg (Fig. 1e and f) of inactivated DI virus + A/WSN. However, mice inoculated with active DI virus + A/WSN remained healthy over this period, showing no clinical signs of disease or weight loss. These data demonstrate that the active DI virus can protect SCID mice against acute disease and that the adaptive immune response plays no significant role over the first few days of the infection.

SCID mice which had been protected from influenza by treatment with 1.2 μg of active DI virus all remained well for 9 days, but on day 10 some started to lose weight and show signs of disease (Fig. 1c and d). The mice developed severe respiratory symptoms and continued weight loss and progressed to death or euthanasia (Fig. 1c and d). SCID mice treated with a higher DI dose (12 μg) remained well for 14 days, but started to lose weight and become ill on day 15 (Fig. 1e and f). At both doses the delayed onset disease had similar dynamics in terms of clinical signs, severity, and duration to the acute influenza seen in SCID mice infected with virus alone. This indicates that the adaptive immune response plays an important role in the late stages of DI virus-mediated protection from influenza virus infection in vivo.

### 3.2 DI virus retards lung consolidation and accumulation of infectious virus load in the lungs of SCID mice.

**Fig. 2:**
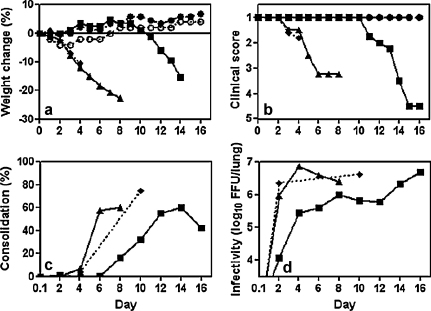
Protection of SCID mice by DI virus monitored by lung infectivity and consolidation. Mice were inoculated with combinations of 1.2 μg of active DI virus + A/WSN or 1.2 μg of inactivated DI virus + A/WSN as described in Fig. 1. Mice were monitored daily for weight change (a), clinical assessment (b), lung consolidation (c) and lung infectivity (d): (■) active DI virus + A/WSN; (▴) inactivated DI virus + A/WSN; (♦) A/WSN alone; (●) active DI virus alone; (○) diluent.

To understand how DI virus mediated protection we examined mice for lung consolidation and lung infectivity. Protection conferred by 1.2 μg of active DI virus (Fig. 2a and b) closely reproduced data shown in Fig. 1. Lungs of SCID mice inoculated with A/WSN only or with inactivated DI virus + A/WSN showed signs of consolidation from day 4 onwards, with lungs exhibiting a plum-coloured discoloration of small areas of the lung surface, particularly around the insertion of the bronchi (Fig. 2c). This looked very similar to the lungs of immune-competent mice infected with A/WSN. Consolidation increased rapidly until, by day 6, the majority of the lung surface was discoloured. During this period there was no sign of consolidation in the lungs of active DI virus-treated, infected mice, but consolidation developed in these animals from day 8. The timing was atypical as the delayed consolidation appeared 3 days before the onset of clinical disease or weight loss instead of 1 to 2 days afterwards seen with the normal acute disease (Table 1). Lung consolidation in active DI virus-treated, virus-infected SCID mice progressed at a similar rate to that in SCID mice given only infectious virus. Consolidation declined in the few active DI virus-treated mice that survived to day 16.

On day 2 post-infection the lung infectivity in SCID mice inoculated with inactivated DI virus + A/WSN was already 10% of the maximum value reached on day 4, while the lung titre in mice receiving active DI virus + A/WSN was 83-fold lower on day 2. Although the infectious load in active DI virus-treated mice increased slowly over the next few days the difference seen with treated with active or inactive DI virus remained at over 10-fold to day 6 post infection. At this time active DI virus-treated, infected mice appeared perfectly normal, while mice that received inactivated DI virus + A/WSN had had lost nearly 20% body mass and were extremely ill. From days 4 to 8 the infectious load in DI treated-mice rose steadily, and at day 8 there was overt lung consolidation (Fig. 2c). Consolidation, infectious virus load, weight loss and clinical disease all increased thereafter (Fig. 2a–d). Taken together, the data show that active DI virus treatment significantly delayed the production of infectious virus in the lungs of SCID mice compared to those treated with inactive DI virus and this correlated with delays in the lung consolidation and overt clinical disease.

### 3.3 The delayed onset disease in active DI virus-treated SCID mice is not due to lung DI RNA declining in amount or undergoing mutation

**Fig. 3:**
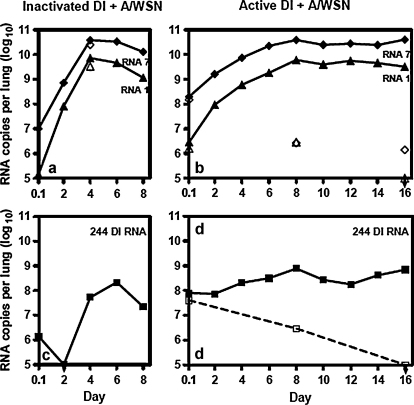
Quantitative RT-PCR of 244 DI RNA and full-length virion RNAs 1 and 7 isolated from the lungs of mice inoculated as described in Fig. 2. Panel (a), mice inoculated intranasally with inactivated DI virus + A/WSN, A/WSN, DI virus, and diluent; panel (b) mice inoculated intranasally with active DI virus + A/WSN. Means of the data from the lungs of 2 replicate mice are shown: (v) 244 DI RNA; (▴) segment 1 virion RNA from mice inoculated with active DI + A/WSN or inactivated DI virus + A/WSN; (♦) segment 7 virion RNA from mice inoculated with active DI + A/WSN or inactivated DI virus + A/WSN. Panel (a) also shows data for mice inoculated with WSN only: segment 1 (▵) and segment 7 (♢). Panels (b) and (d) show data for mice inoculated with DI virus only: segment 1 (▵), segment 7 (♢), and 244 DI RNA (□).

There are no reports in the literature for the dynamics of influenza full-length or DI RNA synthesis in the mouse lung. To determine if the delayed disease was due to changes in the levels of 244 DI or infectious virus RNA, total RNA was isolated from the lungs of each group of SCID mice described in Fig. 2 and subjected to real-time PCR to determine the amounts of 244 DI RNA, genomic segment 1 RNA, and segment 7 RNA (Fig. 3). The levels of segments 1 and 7 RNA on day 2 after infection were similar in the lungs of mice given either inactivated DI virus + A/WSN or active DI virus + A/WSN. On day 4 there was 5-fold less segment 7 and 12-fold less segment 1 in the active DI virus + A/WSN than in the control group but by day 6 both groups had similar amounts of segments 1 and 7. At this point the levels of segments 1 and 7 in the lungs of the inactivated DI virus + A/WSN group reached a plateau, while those in the active DI virus + A/WSN group reached a plateau from day 8. On day 8 mice in the inactivated DI + A/WSN group were very sick indeed, and the amount of RNA in replicate lungs varied by over 100-fold making the mean unreliable. The majority of mice in this group died shortly thereafter. In both groups, levels of segment 7 RNA were consistently 5 to 10-fold greater than those of segment 1. The reasons for this are unclear but as the PCR primers are vRNA specific this appears to be a genuine difference. This is consistent with studies with studies of synchronized infection of cells in vitro in which segment 7 RNA was 9-fold greater than the combined RNAs1 to 3 [36] or 2-fold greater than RNA 1 early in infection [37].

There was an initial high level of approximately 10^8^ copies of 244 DI RNA in the lungs of SCID mice inoculated with the active DI virus + A/WSN, and about 100-fold lower in the group that received inactivated DI virus + A/WSN. The latter represents UV-fragmented 244 RNA and residual intact 244 RNA (Fig. 3c and d). After 2 days there was undetectable 244 DI RNA in the lungs of mice inoculated with inactivated DI virus + A/WSN (Fig. 3c and d), whereas the amount in the active DI virus + A/WSN group was unchanged. 244 RNA in the active DI virus-protected group then maintained a modest but steady rise to nearly 10^9^ copies per lung by day 8, and remained between 10^8^ and 10^9^ copies until day 16 when most of the mice were dead. The RNA was clearly being replicated as mice that received only active DI virus showed a steady decline in amounts of 244 RNA (Fig. 3d open squares). Thus substantial amounts of 244 RNA were present in mice inoculated with DI + A/WSN throughout both the initial period of good health (up to and including day 9) and through the period of delayed onset disease (days 10–16). In contrast 244 DI RNA in the lungs of mice inoculated with inactivated DI virus + A/WSN increased from day 2 to day 4 reflecting rapid replication of residual amounts of DI RNA that remained after the UV-irradiation (Fig. 3c). The 244 RNA increased to a maximum on day 6, but this was evidently too late to be of benefit as 75% of mice already showed signs of clinical disease on day 4.

Although 244 DI RNA was replicated in the lungs of A/WSN-infected SCID mice that were initially protected from acute disease, it was possible that at some stage the 244 DI RNA mutated and was no longer recognised or replicated by the A/WSN ‘helper virus’. To address this, RNA was isolated from the lungs of very sick SCID mice inoculated with DI virus + A/WSN at 16 days post infection. Sequencing confirmed that there were no nucleotide changes compared with the original 244 DI RNA. In addition 5′ and 3′ RACE (rapid amplification of cDNA ends) confirmed that the terminal sequences were also unchanged (data not shown). The same result was obtained in 2 independent experiments, demonstrating that authentic 244 DI was present in substantial amounts in the sick mice on day 16 after infection.

### 3.4 Delayed onset disease in active DI virus-treated SCID mice is not due to challenge virus becoming resistant to DI virus

DI genomes are replicated by the infectious homologous virus and interfere with the production of infectious virus when a critical ratio of DI genomes: infectious genomes is reached. This suggests that there may be evolutionary pressure for the fixation of viral mutations that result in it no longer recognising, replicating or being inhibited by 244 DI RNA. Such resistance has been reported to occur in cell cultures persistently infected by VSV or Sindbis virus [38–44] but not in cells infected with influenza viruses. The latter might be considered unlikely as influenza virus resistant to DI virus would have to develop mutations in each of its 8 independently replicating genome segments.

**Fig. 4:**
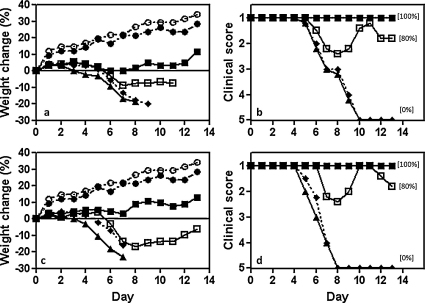
Comparison of the ability of DI virus to protect immune-competent C3H/He-mg mice infected with SCID/WSN-DI virus or the original A/WSN challenge virus. SCID/WSN-DI virus was isolated from SCID mice 16 days after inoculation with active DI virus + A/WSN (see Fig. 2). The latter was given a single passage in MDCK cells and its infectivity titre normalized to that of A/WSN in MDCK cells. Panels (a) and (b), mice infected with SCID/WSN-DI virus; panels (c) and (d), mice infected with A/WSN. Mice were treated with 1.2 μg of active DI virus or inactivated DI virus. Panels (a) and (c), weight change; panels (b) and (d), clinical assessment: (■) 1.2 μg active DI virus + infectious virus; (□) 0.12 μg active DI virus + infectious virus; (▴) 1.2 μg inactivated DI virus + infectious virus; (♦) infectious virus alone; (●) 1.2 μg active DI virus alone; (○) diluent. Other data are as in Fig. 1.

To test this possibility we isolated infectious virus from the lungs of severely ill SCID mice at 16 days after inoculation with active DI virus + A/WSN (Fig. 1). Virus was passed once in MDCK cells (to produce SCID/WSN-DI virus), purified as described in Section 2, and titrated in MDCK cells alongside the original A/WSN challenge virus. The SCID/WSN-DI virus (Fig. 4a and b) was then compared with the original A/WSN challenge virus (Fig. 4c and d) at the same infectivity titre (2.8 × 10^3^ ffu) in an in vivo protection experiment with 244 DI virus and immune competent mice. Data in Fig. 4 show that both viruses had similar virulence when inoculated alone or in the presence of inactivated DI virus, and that 1.2 μg of DI virus gave similar protection to mice infected with SCID/WSN-DI virus or the original A/WSN. A further 10-fold dilution of DI virus gave reduced but still significant protection. This indicates that infectious A/WSN that had been replicating for 16 days in the SCID mice and the original challenge virus recognized 244 DI RNA to a similar extent. Thus the observed breakdown in protection in SCID mice was not due to infectious virus becoming resistant to the DI virus during rounds of multiplication in vivo.

## 4 Discussion

Intranasal inoculation with 244 DI influenza virus completely protected SCID mice from rapid onset acute respiratory disease caused by A/WSN over the period that control groups became severely ill and died. Protected mice appeared completely normal showing no sign of disease or weight loss. This demonstrated that adaptive immune responses, in which SCID mice are totally deficient, are not needed for 244 DI virus-mediated protection. The main correlates of protection from clinical disease and weight loss in mice inoculated with active DI virus + A/WSN compared with control receiving inactivated DI virus + A/WSN are (a) reduction in the amount of infectious virus in the lungs of mice on day 2 (83-fold), day 4 (27-fold) and day 6 (10-fold), (b) reduction in genomic RNAs 1 and 7 in the lung on day 4, (c) larger amounts of 244 DI RNA in the lung on days 2 and 4, and (d) absence of lung consolidation. It appears therefore that the key events necessary to maintain animal wellbeing occur early in infection, with the main protective action of DI virus taking place at 2 and 4 days after infection or earlier. Protection correlated with high amounts of lung DI RNA and low amounts of lung infectivity. Despite the relatively high virus load in the lungs of protected mice, they appeared to be clinically normal at this time, gaining weight, and exhibiting no lung consolidation.

A summary of the main features of the delayed onset disease in SCID mice given the lower dose (1.2 μg) of active 244 DI virus + A/WSN and the acute disease in SCID mice given the same amount of inactivated 244 DI virus + A/WSN is shown in Table 1. In the acute disease, significant weight loss and clinical signs coincided with or occurred 1 day later than infectivity reaching approximately 10^6^ ffu in the lung, with consolidation commencing 1–2 days later. In contrast, mice treated with DI virus attained similar levels of infectivity and significant consolidation on day 8, but significant weight loss and clinical signs were not apparent for another 3 days. However, once initiated the course of disease in the acute and late onset disease groups was indistinguishable. We have not seen any relapse in many hundreds of wild-type mice, with no known immune defect, protected with 244 DI virus from various influenza A viruses, and this includes observing most mice for 7 weeks and some for 6 months after infection (authors’ unpublished data). Lung consolidation in SCID mice infected with an influenza A virus is described as plum coloured areas on the lung surface (as we found), which microscopically presents as a proliferative pneumonia, comprising a massive multifocal to coalescing proliferative bronchitis, bronchiolitis, and alveolitis, marked proliferation of type II pneumocytes, and hyperplastic and hypertrophic columnar epithelium lining the airways [26]. A substantial migration of natural killer cells into the lungs of influenza virus-infected SCID mice has also been reported, although they played no role in disease progression [27]. In mice given a 10-fold higher DI dose, disease was delayed by a further 7 days showing that the delay was DI virus dose-dependent (Fig. 1d and f). Non-clearance of infectivity is the norm in influenza virus-infected SCID mice [28,29,31], not unexpectedly as immune competent mice require B and T cell responses to clear influenza infection [45–47].

Previous work using wild-type mice, A/WSN challenge virus, and non-cloned DI WSN virus showed that there were MHC-restricted virus-specific CD8^+^ and CD4^+^ CTL responses in the lungs of H-2^k^ mice infected with A/WSN or A/WSN + inactivated DI virus. These mice all died. CTL responses were diminished in mice inoculated with A/WSN + DI virus and these all survived [19]. Analysis of the specificity of T cell responses using vaccinia viruses expressing individual influenza A virus proteins showed that, unusually for influenza A virus infections, the response in A/WSN-infected, DI virus-treated mice was largely strain specific. Depletion of both CD8^+^ and CD4^+^ cells with specific antibody was needed to abolish lung consolidation and for mice infected with A/WSN or A/WSN + inactivated DI virus to survive [19], but like the SCID mice reported here, infectious virus in the lung was not cleared. In contrast, when mice depleted of CD8^+^ and CD4^+^ cells were inoculated with A/WSN + DI virus, lung infectivity was cleared, presumably with the assistance of local, T cell-independent, virus-specific antibody. These mice produced a haemagglutinin (HA)-specific antibody that was highly unusual as it was not neutralizing but, when adoptively transferred, protected naïve animals from A/WSN [20,22,25]. The same HA-specific lung IgG conferred cell killing ability on naïve cells in a MHC class I restricted manner [23] In addition, a monoclonal antibody isolated from lung B cells possessed no haemagglutination-inhibition activity but recognised HA on the surface of cells only in the context of the cognate MHC class I antigen, and in so doing mimicked the specificity of a T cell receptor [24]. Thus A/WSN + DI virus stimulated in the lung two highly unusual HA-specific antibodies. Mice infected with A/WSN or A/WSN + inactivated DI virus did not make the HA-specific, non-neutralizing lung antibody. HA-specific antibody from the serum of the same animals was conventionally neutralizing, but evidently did not enter the lung compartment. In summary, there are some unusual and possibly unique interactions between the immune system and DI virus when it is replicated in mice. Broadly it appears that the immunomodulatory activity of influenza A virus is modified by DI virus through its interfering property to produce a generally favourable outcome for the host animal [21]. Whether or not different influenza A DI RNA sequences modulate immune responses in the same way remains to be determined.

Analysis of RNA taken at day 16 from the lungs of sick SCID mice that had received active 244 DI virus + A/WSN showed that the sequence, and thus the properties, of the 244 RNA had not changed. Infectious A/WSN isolated from the same group of mice was also unchanged in sensitivity to interference by 244 DI virus in subsequent tests in immune competent mice in vivo. This lack of acquired resistance to DI virus contrasts with the appearance of oseltamivir-resistant mutants in influenza virus-infected SCID mice [29]. It appears that while adaptive immune responses are not needed for DI-mediated protection from acute disease, they are essential for clearance of infectious virus and, that without such responses, DI virus is unable to prevent disease eventually occurring.

From days 4 to 8 there were small increases in the amounts of infectious virus, genomic RNAs and 244 DI RNA, with all showing a modest peak on day 8, and this build up appears to presage overt late onset disease. The interactive dynamics of infectious virus, genomic RNAs and 244 DI RNA during the initial acute disease/protection phase are difficult to reconcile with the conventional dogma that protection is mediated by the DI RNA competing for replication with cognate full-length segment 1, and thus reducing the amount of infectious virus produced. In fact, we see that on days 2, 4 and 6 after infection, infectivity is lower in the active DI group (by 83-, 27- and 10-fold, respectively) than in the inactivated DI group as expected, but on day 2 both groups had the same level of segment 1. Segment 1 was reduced in the DI group only on day 4 (by 12-fold). In addition to this quantitative disparity, there was no preferential reduction in the cognate segment 1, as segment 7 was reduced in parallel (on day 4 by 5-fold). An intriguing feature of this work was the constant ratio of viral segment 1 RNA: 244 RNA, a segment 1 DI RNA. We saw no evidence of competition for replication between the DI and its cognate full-length RNA segment in the lung. However, we do not know if these data are affected by any asynchronicity of infection of cells by infectious and DI virus, or by heterogeneity of cells in the lung. There is no doubt that DI RNA is being replicated as the amount of DI RNA in lungs of mice inoculated only with DI virus declined by over 100-fold during the experiment. Data show that in the lung segment 1 RNA levels increase faster than lung 244 DI RNA levels and this may explain why there is disease breakthrough. The lowest recorded ratio of segment 1: DI RNA (1.3-fold) occurred on day 2 post infection, with the maximum ratio on day 12 (32-fold). Again there is no preferential difference as the maximum ratio of segment 7: 244 RNA was also on day 12.

Several mechanisms have been proposed for the mode of action of 244 DI virus in vivo including interference with the production of homologous virus via competition between DI and full-length genomes, stimulation of adaptive immune responses, or activation of innate immune responses. The simplest explanation for the disparity between the lung infectious virus load and lung viral genomic RNA is that DI RNA is competing not at the level of RNA replication but at the level of assembly or packaging of virion RNA into new virions. The packaging of influenza virus genome RNA into virus particles is a very specific process with the virus selectively packaging one copy of each of the 8 segments [48]. Thus, packaging of the DI RNA would prevent packaging of the segment from which it was derived and would very efficiently render that virus particle non-infectious. The data presented here also indicate that adaptive immunity is not required for prevention of acute infection in SCID mice but is needed to prevent disease breaking out later. This was not due to genome competition between the segment 1 DI RNA and its cognate full-length segment. In other experiments we have found that 244 RNA fully protects type I interferon receptor null mice from disease resulting from A/WSN infection [49]. However, the possibility that interferon also plays a role in DI-mediated protection of SCID mice has yet to be determined.

## Figures and Tables

**Table 1:** Relationship between lung infectivity and significant levels of clinical disease parameters.

	Lung infectivity of approximately (×10^6^ ffu/lung)	Weight loss	Clinical disease	Lung consolidation
Acute disease^a^	Day 2	Day 2	Day 3	Day 4
Delayed onset disease^b^	Day 8	Day 11	Day 11	Day 8

a In SCID mice inoculated with 1.2 μg inactivated 244 DI virus + A/WSN.

b InSCID mice inoculated with 1.2 μg 244 DI virus + A/WSN.
